# Comparative evidence on the independent contribution of granulocyte-macrophage colony-stimulating factor to anti-GD2 immunotherapy in neuroblastoma: a systematic review and gap analysis

**DOI:** 10.3389/fonc.2026.1932343

**Published:** 2026-09-04

**Authors:** Aleksandra Wieczorek, Gabriela Mielecka-Jarmocik, Anna Synakiewicz, Katarzyna Śladowska, Paweł Kawalec, Holger N. Lode

**Affiliations:** 1 Department of Pediatric Oncology and Haematology, Institute of Pediatrics, Faculty of Medicine, Jagiellonian University Medical College, Krakow, Poland; 2 Department of Pediatrics, Hematology and Oncology, Medical University of Gdansk, Gdansk, Poland; 3 Department of Nutrition and Drug Research, Institute of Public Health, Faculty of Health Sciences, Jagiellonian University Medical College, Krakow, Poland; 4 Department of Pediatric Hematology and Oncology, University Medicine Greifswald, Greifswald, Germany

**Keywords:** anti-GD2 immunotherapy, comparative effectiveness, granulocyte-macrophage colony-stimulating factor, neuroblastoma, systematic review

## Abstract

**Objective:**

To assess comparative evidence on the independent contribution of GM-CSF to the efficacy and safety of anti-GD2 immunotherapy in neuroblastoma and to identify evidence gaps.

**Methods:**

This systematic review was conducted in accordance with the PRISMA guidelines in February 2026. MEDLINE (via PubMed), EMBASE, and the Cochrane Library were searched to identify comparative studies evaluating anti-GD2 immunotherapy with and without GM-CSF in patients with neuroblastoma. In addition, the websites of key oncology societies were searched.

**Results:**

No study directly compared otherwise equivalent anti-GD2 regimens that differed only in the inclusion of GM-CSF. Two studies met the broader comparative eligibility criterion and provided only indirect regimen-level information. Both evaluated older, non-licensed murine anti-GD2 antibodies (m3F8 and 14.G2a), and neither evaluated GM-CSF with currently licensed anti-GD2 antibodies. In the retrospective study by Cheung et al., survival outcomes differed across treatment eras; however, GM-CSF was co-administered with 13-cis-retinoic acid and treatment groups also differed with respect to prior stem cell transplantation, induction therapy, baseline characteristics, follow-up duration and supportive care. Therefore, the observed survival differences cannot be attributed to GM-CSF. Toxicity comparisons in this study were also limited by differences in toxicity ascertainment across treatment eras. The prospective phase I/Ib study by Frost et al. was primarily designed to evaluate dose finding and toxicity, included very small and heterogeneous treatment groups, and did not isolate GM-CSF as an independent treatment variable. No eligible comparative study was identified for dinutuximab, dinutuximab beta or naxitamab.

**Conclusion:**

Available comparative evidence is insufficient to determine the independent contribution of GM-CSF to the efficacy or safety of anti-GD2 immunotherapy in neuroblastoma. These findings should not be used to justify modification of licensed product-specific anti-GD2 regimens. A randomized trial holding the anti-GD2 backbone and relevant co-interventions constant is needed to isolate the effect of GM-CSF.

**Systematic Review Registration:**

https://www.crd.york.ac.uk/PROSPERO/, identifier CRD420251116911.

## Highlights

- GM-CSF is included in several anti-GD2 regimens, but its independent contribution remains uncertain.
- Only two eligible comparative studies were identified; both evaluated historical, non-licensed murine anti-GD2 antibodies.
- No eligible comparative study evaluated the contribution of GM-CSF to immunotherapy with dinutuximab, dinutuximab beta, or naxitamab.
- Observed survival and toxicity differences in historical studies could not be attributed specifically to GM-CSF.
- Current evidence does not support modifying licensed product-specific regimens; prospective evaluation is warranted.

## 1 Introduction

Neuroblastoma is a malignant tumor arising from embryonal cells committed to the development of the sympathetic nervous system. It is the most common extracranial solid tumor in children, with a median age at diagnosis of 17 months, and accounts for approximately 15% of pediatric cancer-related deaths (1, 2).

The prognosis for patients with neuroblastoma varies widely depending on several factors, primarily the child’s age at diagnosis, disease stage, the presence of specific genetic mutations such as *MYCN* amplification, and tumor histology (3). Patients with stage M disease (stage 4 according to the International Staging System [INSS]) who are older than 18 months at diagnosis, as well as those with *MYCN*-amplified neuroblastoma beyond stage 1 INSS, are considered as having high-risk disease (3). This group is associated with a poor prognosis and a 5-year survival rate of approximately 50% (4, 5).

Treatment of high-risk neuroblastoma (HR-NBL) is intensive and multimodal, typically involving induction chemotherapy, surgery, radiation therapy, high-dose chemotherapy with autologous stem cell transplantation (SCT), immunotherapy, and retinoid therapy. Most neuroblastoma cells express high levels of disialoganglioside-2 (GD2), which is targeted by several monoclonal antibodies currently used in therapy. Anti-GD2 immunotherapy and chemoimmunotherapy have demonstrated efficacy in HR-NBL, both in newly diagnosed patients and in those with relapsed disease (1, 5–21).

Three anti-GD2 antibodies have received regulatory approval for clinical use: ch14.18/SP2/0 (dinutuximab), a ch14.18 chimeric antibody produced in murine cells and approved by the U.S. Food and Drug Administration (FDA) under the brand name Unituxin^®^ (22); ch14.18/CHO antibody (dinutuximab beta), a mouse–human chimeric monoclonal immunoglobulin G1 antibody produced in Chinese hamster ovary (CHO) cells and approved by the European Medicines Agency under the brand name Qarziba^®^ (23); and hu3F8 antibody (naxitamab), a humanized antibody produced in CHO cells and approved by the FDA under the brand name Danyelza^®^ (24, 25). Murine monoclonal antibodies 3F8 and 14.G2a, previously used in clinical trials, are not approved for clinical use.

Anti-GD2 immunotherapy has been investigated in clinical trials in HR-NBL in combination with cytokines. A phase 3 randomized controlled trial (RCT; ANBL0032) evaluating the clinical efficacy of dinutuximab in combination with GM-CSF and interleukin 2 (IL-2), added to differentiation therapy with isotretinoin (13-cis-RA), demonstrated significantly improved survival outcomes (7, 11). While the effectiveness of anti-GD2 therapy was well established in this comparative study, the specific contribution of concurrently administered agents remained unclear. Results from a phase 3 RCT (HRNBL-1/SIOPEN) showed that the addition of subcutaneous IL-2 to dinutuximab beta did not improve outcomes in newly diagnosed patients with HR-NBL who had responded to standard induction and consolidation therapy (26). Furthermore, the combination of IL-2 with dinutuximab beta was associated with increased toxicity compared to dinutuximab beta alone (26). A lack of additional benefit from IL-2 was also demonstrated in an RCT in patients with relapsed or refractory HR-NBL (27).

In the maintenance phase, anti-GD2 immunotherapy is administered with isotretinoin, which is included in the treatment regimen because of its pro-differentiating mechanism of action. There are no comparative studies evaluating anti-GD2 immunotherapy with and without isotretinoin; however, the evidence supporting the independent contribution of isotretinoin in the context of modern anti-GD2-based maintenance therapy remains uncertain (28). Dinutuximab beta has also been studied as a single agent in patients with relapsed or refractory disease, with survival outcomes comparable to those observed when it is administered with cytokines and isotretinoin (10, 29). However, comparative evidence regarding the benefit of adding isotretinoin to anti-GD2 immunotherapy is lacking.

The results of the above studies underscore the importance of not only evaluating the addition of new therapies but also reassessing the necessity of existing treatments that may impose an unnecessary burden on patients (30). This consideration is particularly important for patients with HR-NBL, who are subjected to prolonged and highly intensive therapeutic regimens.

Granulocyte-macrophage colony-stimulating factor (GM-CSF) is a cytokine that stimulates the bone marrow to produce white blood cells, particularly granulocytes and macrophages. It plays an important role in the immune system by promoting the proliferation and differentiation of these cells, which is essential for host defense against infections. Its original clinical application was the treatment of neutropenia following chemotherapy or bone marrow transplantation (BMT). However, because of a higher toxicity profile compared with granulocyte colony-stimulating factor (G-CSF), it is not routinely used for these indications. GM-CSF can modulate the functions of innate immune cells and activate adaptive immune responses, potentially influencing cancer development and progression (31). In addition, GM-CSF has been shown to enhance cytotoxicity against neuroblastoma cells through several pathways. It enhances anti-GD2 monoclonal antibody (MoAb)–dependent cell cytotoxicity and increases the phagocytosis of neuroblastoma cells by activated macrophages (32, 33). On the other hand, GM-CSF may also negatively affect the efficacy of anticancer therapies by promoting immune cell exhaustion, increasing regulatory T cells, enhancing angiogenesis, reducing tumor antigen presentation, and favoring a tumor-promoting macrophage phenotype (31).

The use of anti-GD2 antibodies with or without GM-CSF is largely determined by the design of the clinical trials on which their marketing authorization was based. Dinutuximab and naxitamab are indicated for use in combination with GM-CSF (22, 24, 25), whereas dinutuximab beta is approved for use without GM-CSF (23). However, the necessity of combining GM-CSF or G-CSF with anti-GD2 therapies remains unclear and continues to be debated (32, 33). Therefore, the aim of this systematic review was to systematically and critically appraise the comparative evidence regarding the independent contribution of GM-CSF to anti-GD2 immunotherapy in patients with neuroblastoma and identify evidence gaps.

## 2 Materials and methods

A systematic review was conducted in accordance with the Preferred Reporting Items for Systematic Reviews and Meta-Analyses (PRISMA) guidelines (34) and the Cochrane Handbook for Systematic Reviews of Interventions (35). The review was registered in the PROSPERO database (registration number: CRD420251116911) (36). The literature search was conducted on February 12, 2026, using the MEDLINE (via PubMed), EMBASE, and Cochrane Library databases. The search strategy was based on Medical Subject Headings (MeSH) combined with Boolean logical operators. The detailed search strategy is presented in **Supplementary Table 1**. The reference lists of the included studies and the websites of three oncology societies—the American Society of Clinical Oncology (ASCO), the European Society for Medical Oncology (ESMO), and the International Society of Paediatric Oncology (SIOP)—were also searched (**Supplementary Table 4**).

The eligibility criteria were prespecified according to the PICOS framework (Population, Intervention, Comparator, Outcomes, and Study design) and are presented in **Table 1**. The review question concerned the component-specific contribution of GM-CSF rather than the overall activity of GM-CSF-containing regimens. Accordingly, studies were eligible for evidence synthesis only if they included an anti-GD2-treated group receiving GM-CSF and an anti-GD2-treated comparator group not receiving GM-CSF. Single-arm studies can characterize regimen-level activity and safety but cannot distinguish the contribution of GM-CSF from that of the anti-GD2 antibody, concomitant therapy, patient selection, prior treatment, or the natural history of disease. Selected contemporary single-arm studies were summarized only to provide clinical context and were not used to infer a component-specific effect. The prespecified population was restricted to patients with neuroblastoma; however, a single *post hoc* exception was made for Frost et al., whose cohort included 31 patients with neuroblastoma and 2 patients with osteogenic sarcoma. Because the latter patients could not be excluded from all pooled outcome data, the study was retained solely as indirect regimen-level evidence, and this deviation from the prespecified eligibility criteria was acknowledged as a limitation of the review.

**Table 1:** Inclusion and exclusion criteria for the systematic review.

Eligibility criterion	Inclusion criteria	Exclusion criteria
Population	Patients of any age with neuroblastoma. Mixed-tumor cohorts were eligible when neuroblastoma-specific data were reported separately; exceptionally, a mixed cohort in which neuroblastoma was the predominant diagnosis could be retained as indirect evidence when patients with other tumors could not be fully excluded from the reported outcomes	Patients with tumors other than neuroblastoma; mixed-tumor cohorts without separately reported neuroblastoma data, except for the single documented *post hoc* Frost et al. exception described in the Methods and table footnote
Intervention	GM-CSF used in combination with any anti-GD2 therapy; use of other cytokines allowed	GM-CSF not used in combination with anti-GD2 therapy
Comparator	Anti-GD2 therapy without GM-CSF; use of other cytokines allowed	None
Outcomes	Efficacy (survival outcomes and/or treatment response) Quality of life Safety	Trials not reporting defined outcomes
Study design	Any comparative study including at least one group treated with an anti-GD2 regimen containing GM-CSF and at least one comparator group treated with an anti-GD2 regimen without GM-CSF. Because direct comparisons were expected to be scarce, studies in which other treatment components differed between groups were eligible but were interpreted only as indirect regimen-level evidence	Non-comparative studies, including single-arm studies and case reports; reviews and letters
Publication type	Published full-text articles and conference abstracts*	Editorials, letters, data from clinical trial registries
Language	Any language	None

GM-CSF, granulocyte-macrophage colony-stimulating factor. *Conference abstracts identified through oncology society websites were screened systematically using the same eligibility criteria as full-text publications. Abstract-only records were eligible for inclusion if they provided sufficient information to establish the presence of an anti-GD2 plus GM-CSF arm and a comparator arm without GM-CSF and reported at least one predefined efficacy or safety outcome. *Post hoc* deviation: Frost et al. enrolled 31 patients with neuroblastoma and 2 with osteogenic sarcoma. Because the non-neuroblastoma cases could not be fully excluded from all pooled outcomes, the study was retained only as indirect regimen-level evidence.

Titles and abstracts of all identified records were screened independently by two review authors (K.Ś. and P.K.). Full texts of potentially eligible reports were retrieved and independently assessed by the same review authors against the predefined eligibility criteria. Disagreements at either stage were resolved through discussion and consensus.

Data from the included studies were extracted independently by two review authors (K.Ś. and P.K.) using a predefined standardized data-extraction form. Discrepancies were resolved through discussion and consensus. The extracted data included study design, sample size, participant characteristics, treatment regimens, treatment and follow-up durations, and outcomes of interest.

Risk of bias was assessed independently by two review authors, with disagreements resolved by consensus, using the original ROBINS-I tool (2016) (37). Assessments were performed separately for each outcome across the seven ROBINS-I domains, with overall judgments classified as low, moderate, serious, critical, or no information. The target effect was defined as the effect of assignment to GM-CSF in addition to an otherwise equivalent anti-GD2 regimen versus assignment to the same regimen without GM-CSF. Treatment era, baseline disease status and prognostic factors, induction or salvage therapy, stem-cell transplantation, isotretinoin, anti-GD2 dose, and IL-2 dose and schedule were identified *a priori* as potential confounders for the ROBINS-I assessment. Differences in follow-up and outcome-ascertainment procedures were evaluated in the relevant ROBINS-I domains. Detailed domain-level judgments and supporting rationales are presented in **Supplementary Table 5**.

Where a reported percentage was inconsistent with the stated numerator and denominator, the percentage was recalculated from the reported counts, and the discrepancy was explicitly noted. After evaluating clinical and methodological heterogeneity, the feasibility of conducting a meta-analysis or a formal indirect comparison was assessed. Because a valid component-specific quantitative synthesis was not feasible, the results were synthesized descriptively and presented in narrative form, study-level tables, and a schematic comparison of treatment components. No formal subgroup or sensitivity analyses were conducted. Because no quantitative synthesis was performed, no separate synthesis-level assessment of reporting bias due to missing results was undertaken; within-study selective reporting was evaluated in ROBINS-I domain 7. No formal certainty-of-evidence assessment was undertaken.

## 3 Results

### 3.1 Search results and general information about included studies

No study directly compared otherwise equivalent anti-GD2 regimens that differed only in the inclusion of GM-CSF. A total of 1,631 potentially relevant records were identified: 966 from the bibliographic databases and 665 from the websites of key oncology societies. Of the database records, 28 full-text reports were assessed for eligibility. None of the 665 society-website records met the eligibility criteria. Overall, two studies were included.

Several contemporary studies evaluating GM-CSF-containing anti-GD2 regimens were identified during screening; however, they were single-arm or otherwise non-comparative studies without a comparator group receiving anti-GD2 therapy without GM-CSF and therefore did not meet the predefined eligibility criteria; selected studies are summarized in **Supplementary Table 7** (38–42).

**Figure 1:**
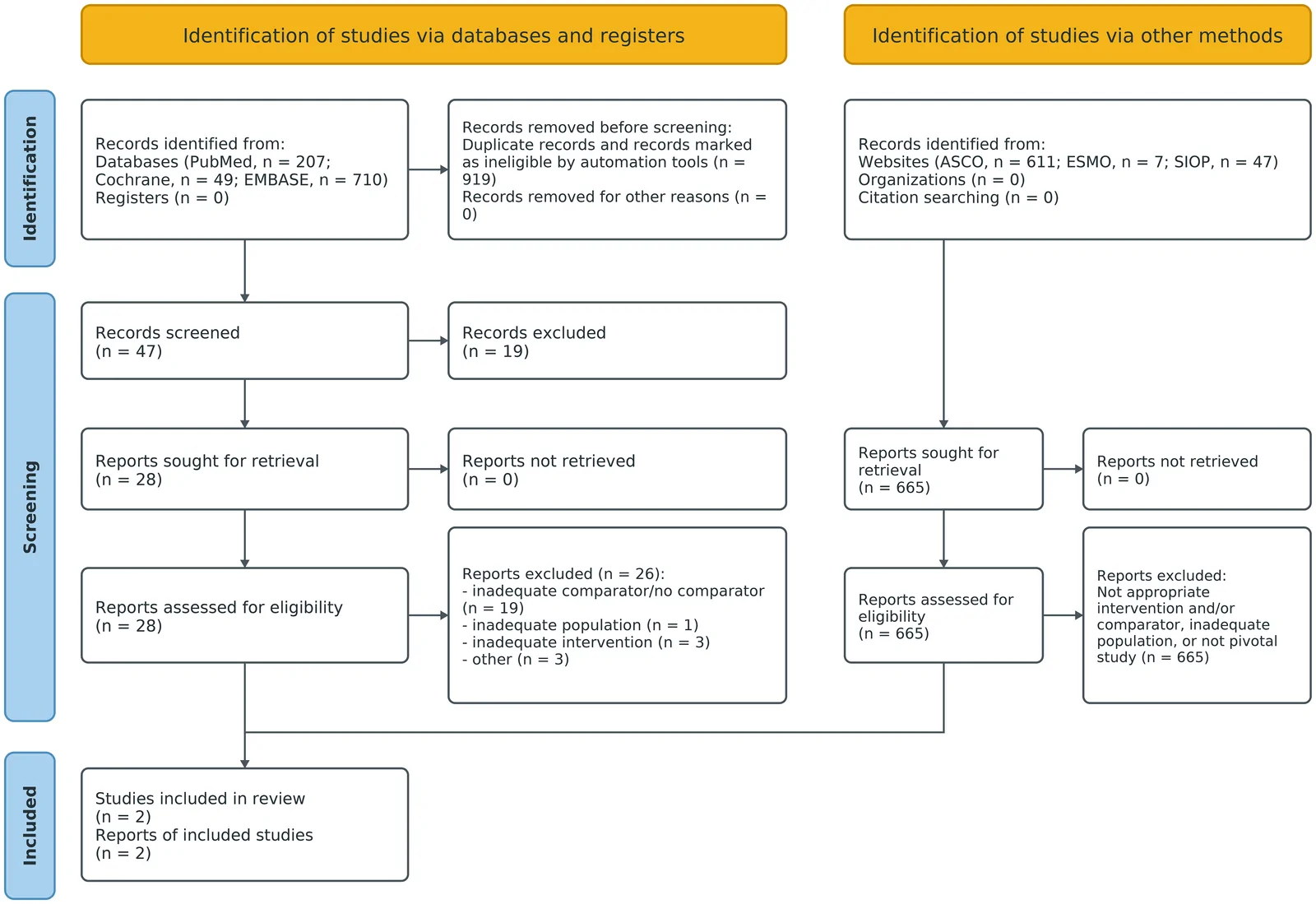
PRISMA 2020 flow diagram of the study-selection process. Adapted from (34).

Two studies met the broader eligibility criterion and provided indirect regimen-level comparative information: Cheung et al. (2012) (43) and Frost et al. (1997) (44) (**Figure 1**).

**Figure 2:**
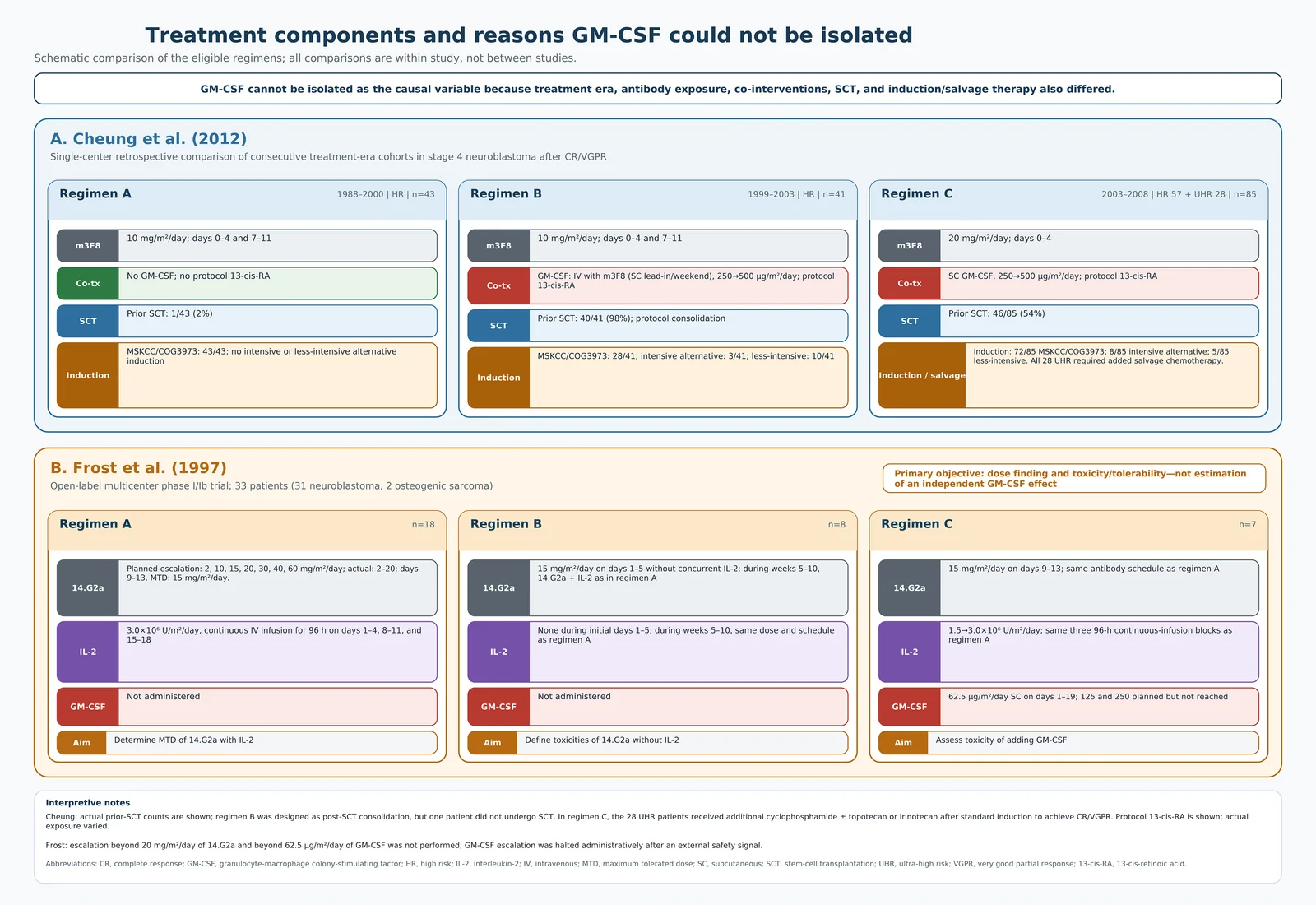
Treatment components in the two eligible studies and reasons why GM-CSF could not be isolated as the causal variable. Upper panel (Cheung et al.) displays regimens A, B, and C by m3F8 dose and schedule, GM-CSF route, 13-cis-retinoic acid, prior SCT, treatment era, and major induction or salvage differences. Lower panel (Frost et al.) displays regimens A, B, and C by 14.G2a dose, GM-CSF exposure, IL-2 dose and schedule, sample size, and the phase I/Ib objective. Comparisons are intended within each study; the two studies are not directly comparable. GM-CSF, granulocyte-macrophage colony-stimulating factor; IL-2, interleukin-2; IV, intravenous; MTD, maximum tolerated dose; SC, subcutaneous; SCT, stem-cell transplantation; 13-cis-RA, 13-cis-retinoic acid.

The study designs and treatment regimens, baseline patient characteristics, efficacy outcomes, and safety outcomes are summarized in **Supplementary Tables 8–12**. A schematic comparison of the principal treatment components across the eligible study arms is presented in **Figure 2**.

Cheung et al., 2012 (43) was an open-label, single-center comparative study based on retrospectively collected data from children with stage 4 HR-NBL. Patients received chemotherapy based on the Memorial Sloan-Kettering Cancer Center (MSKCC) or COG3973 induction regimens, followed by hyperfractionated local radiotherapy with 21 Gy and surgical resection of the primary tumor. Some patients also received second-line therapy to achieve remission and/or myeloablative therapy with stem cell rescue. Patients with ultra–high-risk neuroblastoma, defined as refractory disease after induction, who required additional cyclophosphamide with or without topotecan or irinotecan after standard induction therapy to achieve a complete response (CR) or very good partial response (VGPR) were considered eligible for immunotherapy with the murine anti-GD2 antibody 3F8 (m3F8). Following the achievement of CR or VGPR, patients were treated with one of the three regimens, depending on the year their treatment was initiated:

- regimen A (n=43): m3F8 monotherapy;
- regimen B (n = 41): m3F8 combined with intravenous GM-CSF and 13-cis-retinoic acid in a protocol designed as post-SCT consolidation; 40 of 41 patients had undergone SCT;
- regimen C included 85 patients (57 HR and 28 UHR); 46 of the 85 had undergone SCT. All patients received m3F8 combined with subcutaneous GM-CSF and 13-cis-retinoic acid. The 28 UHR patients had refractory disease after induction and required additional chemotherapy to achieve CR or VGPR before immunotherapy.

All patients without disease progression had a minimum follow-up of 2.9 years from the initiation of immunotherapy and at least 3.6 years from the time of diagnosis. The assessed outcomes included survival metrics, relapse rates, safety profiles of the treatment regimens, and prognostic variables.

The study by Frost et al. (1997) (44) was an open-label, multicenter, prospective phase I/Ib trial conducted in patients younger than 21 years with biopsy-proven neuroblastoma or osteogenic sarcoma. The final cohort comprised 31 patients with neuroblastoma and 2 with osteogenic sarcoma. Although the study did not isolate GM-CSF as an independent treatment variable and was designed primarily to evaluate dose finding and toxicity, it was retained under a *post hoc* mixed-cohort exception because patients with neuroblastoma constituted most of the study population and the 2 osteogenic sarcoma cases could not be separated from all pooled outcomes. Its findings were therefore interpreted only as indirect regimen-level evidence. Eligible patients had failed to achieve a CR following aggressive chemotherapy, including autologous BMT; had progressive disease after such therapy; or had a partial response to reinduction therapy but remained at high risk of tumor progression. All participants were required to have received at least one prior multi-agent chemotherapy regimen. Additional eligibility criteria included an Eastern Cooperative Oncology Group performance status of 0–2, a life expectancy of at least 3 months, and adequate organ and hematologic function. The aim of the study was to assess the toxicity and tolerability of escalating doses of the murine monoclonal antibody 14.G2a (currently not approved for clinical use) administered in combination with IL-2 and GM-CSF (44).

Patients receiving regimen A (n=18) were treated with 14.G2a in combination with IL-2. To determine the maximum tolerated dose (MTD) of 14.G2a, the antibody was administered at escalating doses of 2, 10, 15, 20, 30, 40, and 60 mg/m^2^/day as a 2-hour intravenous infusion for 5 consecutive days (days 9–13 of the treatment course). Regimen B (n=8) was designed to assess toxicities specific to 14.G2a administered without IL-2 at the MTD identified in regimen A. The MoAb was administered at 15 mg/m^2^/day on days 1–5 without concomitant IL-2. During weeks 5–10, all patients received 14.G2a with IL-2, following the same schedule as in regimen A. Patients receiving regimen C (n=7) underwent evaluation of the toxicity profile associated with escalating doses of GM-CSF added to the MoAb and IL-2 treatment schedule. Four dose combinations were planned, with 3 patients assigned to each group. All patients followed the same 14.G2a and IL-2 schedule as in regimen A (15 mg/m^2^/day of 14.G2a), with the addition of subcutaneous GM-CSF administered on days 1–19. In the first group, IL-2 was administered at a dose of 1.5 × 10^6^ U/m^2^/day and GM-CSF at 62.5 µg/m^2^/day. In the second group, the IL-2 dose was increased to 3.0 × 10^6^ U/m^2^/day, while the doses of 14.G2a and GM-CSF remained unchanged. In the third and fourth groups, the GM-CSF dose was planned to be escalated to 125 and 250 µg/m^2^/day, respectively, with IL-2 and 14.G2a doses unchanged. However, escalation was administratively halted at 62.5 µg/m^2^/day based on a safety signal from a separate study in metastatic renal cell carcinoma in which GM-CSF was co-administered with IL-2 (44).

No information was provided regarding the mean or median duration of follow-up. Approximately 30.3% of patients had a history of total body irradiation and BMT. The primary outcomes assessed included toxicity profiles and the development of anti-drug antibodies. No pooled survival outcomes were evaluated, and only limited survival data were available for individual patients (44).

Patient characteristics for the included studies are presented in **Supplementary Table 9**. In the study by Cheung et al. (2012) (43), among high-risk patients, a higher proportion of *MYCN* amplification was observed in those treated with GM-CSF, as well as a higher proportion of patients who received autologous stem cell transplantation (ASCT) and 13-*cis*-RA. However, a statistical comparison between the subgroups was not reported. In the study by Frost et al. (1997) (44), patient characteristics were reported only for the overall population and were not presented separately for patients treated with and without GM-CSF.

Both studies were judged to be at critical overall risk of bias for estimating the independent effect of GM-CSF. In Cheung et al., critical confounding arose from treatment-era differences and systematic imbalances in co-interventions and prognostic factors, with additional concerns regarding non-uniform safety ascertainment. The later treatment eras of the GM-CSF-containing cohorts, together with greater institutional experience and changes in supportive and anticancer care, may have biased the observed outcomes in favor of GM-CSF. Conversely, the poorer-prognosis composition of regimen C, including the ultra-high-risk subgroup, and the higher prevalence of MYCN amplification among GM-CSF-treated high-risk patients could have biased the estimated effect against GM-CSF; therefore, the net direction of confounding was unpredictable.

In Frost et al., the phase I/Ib dose-escalation design, non-equivalent antibody and IL-2 exposure, very small groups, and outcome reporting not designed for a GM-CSF contrast precluded a credible causal estimate. Domain-level judgments are presented in **Supplementary Table 5**.

A reliable meta-analysis of the studies by Cheung et al. (43) and Frost et al. (44) was considered unfeasible because of substantial heterogeneity in study designs, patient populations, interventions, and assessed endpoints.

### 3.2 Efficacy analysis from included studies

Cheung et al. (2012) (43) reported significant differences in 5-year progression-free survival (PFS) (*P* = 0.018) and overall survival (OS) in a univariate analysis comparing all four patient groups: regimen A, regimen B, and regimen C (subdivided into high-risk and ultra–high-risk subgroups). The 5-year PFS was 44% in patients treated with regimen A (i.e., m3F8 without GM-CSF and 13-*cis*-RA), compared with 56% in those receiving regimen B (m3F8 + intravenous GM-CSF + 13-*cis*-RA) and 62% in high-risk patients treated with regimen C (m3F8 + subcutaneous GM-CSF + 13-*cis*-RA; 50% in the subpopulation without SCT). The 5-year OS was 49% in patients treated with regimen A, as compared with 61% in patients treated with regimen B and 81% in high-risk patients treated with regimen C (84% in the subpopulation without SCT). In this context, PFS may be less affected than OS by subsequent lines of therapy and may therefore more closely reflect the initial treatment period, whereas OS, although the most definitive survival endpoint, also captures the effects of post-progression treatments. Although higher survival rates were observed in regimens containing GM-CSF and 13-cis-RA, the comparison is heavily confounded by differences in treatment era, prior SCT, 13-cis-RA exposure, induction therapy, and baseline patient characteristics. Consequently, the observed survival differences cannot be attributed specifically to GM-CSF. Moreover, it is not possible to determine whether the observed outcomes were related to GM-CSF, 13-cis-RA, prior SCT, or their combined effects. Therefore, these findings should be interpreted with considerable caution. Limitations of the study are discussed in detail in the Discussion section.

The relapse rate was highest in regimen A (53%; median follow-up, 15.5 years), followed by regimen B (41%; median follow-up, 10.2 years) and regimen C (37% in the high-risk subgroup; median follow-up, 5.9 years). Patients from the ultra–high-risk subgroup of regimen C had notably poorer outcomes, with a 5-year PFS of 36% and a 5-year OS of 75% (**Supplementary Table 10**).

The efficacy results from the study by Frost et al. (1997) (44) are inconclusive because of the small number of patients with evaluable disease, the reporting of data for all patients combined, and the study’s primary objective being the assessment of regimen toxicity (**Supplementary Table 10**). Among the 26 patients who completed one full treatment course, 9 had no disease progression during the evaluation period (9/26, 35%). The source article reported this proportion as 27%, apparently using all 33 treated patients as the denominator (9/33, 27%); all 7 patients in the GM-CSF group experienced disease progression. Four patients with neuroblastoma from regimen A treated with anti-GD2 immunotherapy and IL-2 demonstrated either clinical or bone marrow responses during treatment. No responses were observed in patients with neuroblastoma treated with regimen B (anti-GD2 + IL-2) or regimen C (anti-GD2 + IL-2 with the addition of GM-CSF).

### 3.3 Safety analysis from included studies

In Cheung et al. (2012) (43), the reported frequencies of individual grade 3–4 toxicities differed across regimens (**Supplementary Table 11**). However, these frequencies were not directly comparable because toxicity ascertainment and recording procedures differed across treatment eras. Within regimen A, only the 24 patients treated under the N7 protocol had toxicity monitoring comparable to that used for the later cohorts after implementation of the MSKCC clinical database. Consequently, differences in the reported frequencies or counts of toxicities may reflect changes in ascertainment and reporting rather than true differences between treatment regimens and cannot be used to estimate the independent effect of GM-CSF on severe toxicity.

In the study by Frost et al. (1997) (44), IL-2 was included in all three regimens; however, in regimen B, 14.G2a was initially administered alone on days 1–5 before subsequent combination treatment with IL-2. The rate of grade 2 or 3 toxicities per treatment course was numerically higher in patients treated with GM-CSF (regimen C) than in those treated without GM-CSF (regimens A and B), whereas the rate of grade 4 toxicities was lower in the GM-CSF group (**Supplementary Table 12**). Toxicities attributed to 14.G2a included pain, allergic or anaphylactic reactions, and rash; however, the small number of patients in regimen C limited meaningful safety comparisons. GM-CSF, administered at doses below the threshold for myelostimulation, did not appear to increase the incidence of severe toxicities, although the incidence of grade 2 or 3 toxicities was higher in patients treated with this cytokine than in the other groups. Because of the limited sample size and the relatively low GM-CSF dosing in regimen C, no conclusions could be drawn regarding its potential to modify the biologic effects of 14.G2a.

## 4 Discussion

No study directly compared otherwise equivalent anti-GD2 regimens that differed only in the inclusion of GM-CSF. Two studies provided indirect comparative information under the broader eligibility criterion; both evaluated historical, non-licensed murine anti-GD2 antibodies, and both contained major differences between treatment groups beyond GM-CSF. Accordingly, these studies provide only indirect regimen-level evidence and cannot estimate the independent effect of GM-CSF.

Cheung et al. compared consecutive treatment cohorts from different eras in a retrospective analysis. GM-CSF was introduced together with 13-cis-retinoic acid, while the cohorts also differed in stem-cell transplantation exposure, induction therapy, baseline prognostic characteristics, anti-GD2 administration, follow-up, supportive care, and toxicity ascertainment. Because GM-CSF and 13-cis-retinoic acid were always co-administered and no analysis could control the full set of time-varying differences, the reported survival and safety differences cannot be attributed specifically to GM-CSF. Restriction to patients in CR or VGPR and the use of murine m3F8 further limit the applicability of these findings to currently licensed antibodies (43).

The study by Frost et al. was a small, open-label phase I/Ib sequential dose-escalation study designed to determine the toxicity and maximum tolerated dose of 14.G2a-based therapy, rather than the independent effect of GM-CSF. The regimens differed in 14.G2a dose, IL-2 schedule, treatment timing, and GM-CSF exposure, and the study included a small and heterogeneous population. Consequently, the reported efficacy and toxicity differences do not provide a credible estimate of the independent contribution of GM-CSF (44).

More broadly, differences between the historical murine antibodies and currently licensed chimeric or humanized antibodies in Fc interactions, immunogenicity, dosing, treatment setting, and co-interventions preclude direct extrapolation of the observed regimen-level outcomes to dinutuximab, dinutuximab beta, or naxitamab (5, 45, 46).

Preclinical data provide biological plausibility for combining GM-CSF with anti-GD2 therapy. GM-CSF may activate granulocytes and monocyte-macrophages and enhance antibody-dependent cytotoxicity or phagocytosis; however, these effects may vary with dose, route, and the tumor microenvironment, and immunosuppressive effects have also been described (31, 32, 47, 48). G-CSF may likewise enhance neutrophil-mediated cytotoxicity (33), but no comparative clinical evidence establishes equivalence, superiority, or lower net toxicity in this setting. Mechanistic plausibility should therefore not be interpreted as proof of a component-specific clinical benefit.

GM-CSF is included in several dinutuximab- and naxitamab-based regimens used in North American practice, whereas current SIOPEN dinutuximab beta regimens do not include concomitant cytokines (12, 17, 18, 22, 24, 32).

Because dinutuximab and naxitamab are indicated in GM-CSF-containing regimens, whereas dinutuximab beta is authorized without GM-CSF, the absence of evidence for an independent GM-CSF effect should not be used to justify deviation from approved product-specific regimens (22–24).

This review is limited by the scarcity and nature of the available evidence. Only two historical studies met the broadened eligibility criterion, and neither compared otherwise equivalent regimens. Their populations, antibodies, co-interventions, treatment eras, follow-up periods, and outcome definitions were heterogeneous, which precluded quantitative synthesis. The Frost cohort included two patients with osteogenic sarcoma, and some outcomes were reported for the mixed-tumor cohort. In addition, the outcome-specific ROBINS-I judgments were constrained by incomplete reporting in the primary studies. These limitations restrict inference to identifying an evidence gap rather than estimating a treatment effect.

### 4.1 Clinical implications

Because no comparative study isolated GM-CSF with a licensed antibody, this review does not support either adding GM-CSF to, or removing it from, any approved regimen. Instead, the findings define equipoise for prospective evaluation of GM-CSF omission, substitution, dose, route or schedule while holding the antibody backbone and relevant co-interventions constant.

## 5 Conclusions

Available comparative evidence is insufficient to determine the independent contribution of GM-CSF to anti-GD2 immunotherapy in neuroblastoma. Both eligible studies evaluated historical, non-licensed murine antibodies and were at critical risk of bias for a GM-CSF-specific causal effect. The findings therefore support neither removing GM-CSF from regimens licensed with it nor adding GM-CSF to regimens licensed without it. A prospective randomized controlled evaluation in which the anti-GD2 backbone and relevant co-interventions are held constant is needed to assess GM-CSF omission, addition, dose, route, or schedule.

## Data Availability

The original contributions presented in the study are included in the article/**Supplementary Material**. Further inquiries can be directed to the corresponding author.
