# Supplementary material for: Comparative evidence on the independent contribution of granulocyte-macrophage colony-stimulating factor to anti-GD2 immunotherapy in neuroblastoma: a systematic review and gap analysis
Source: Front Oncol. 2026 Sep 4;16:1932343. doi: 10.3389/fonc.2026.1932343 (PMC13585510; doi:10.3389/fonc.2026.1932343)
Supplement: Supplementary file 1 [file Table1.docx]

# **Supplement**

**Supplementary Tables 1–4 and 6–7;**

**Supplementary Table 1. PubMed – last search: 12.02.2026.**

| **Number** | **Keywords** | **Search results** |
| --- | --- | --- |
| **#1** | Neuroblastoma OR NBL OR neuroblastomas OR Ganglioneuroblastoma OR Esthesioneuroblastoma | 53 714 |
| **#2** | Granulocyte-Macrophage Colony-Stimulating Factor OR Granulocyte macrophage Colony Stimulating Factor OR GM-CSF OR GM CSF OR CSF-GM OR Histamine-Producing Cell-Stimulating Factor OR Histamine Producing Cell Stimulating Factor OR GM Colony-Stimulating Factor OR CSF-2 OR TC-GM-CSF OR Molgramostin OR Sargramostim | 36 949 |
| **#3** | **#1 AND #2** | 207 |

**Supplementary Table 2. Cochrane Library - last search: 12.02.2026 (all text).**

| **Number** | **Keywords** | **Search results** |
| --- | --- | --- |
| **#1** | Neuroblastoma OR NBL OR neuroblastomas OR Ganglioneuroblastoma OR Esthesioneuroblastoma | 717 |
| **#2** | Granulocyte-Macrophage Colony-Stimulating Factor OR Granulocyte macrophage Colony Stimulating Factor OR GM-CSF OR GM CSF OR CSF-GM OR Histamine-Producing Cell-Stimulating Factor OR Histamine Producing Cell Stimulating Factor OR GM Colony-Stimulating Factor OR CSF-2 OR TC-GM-CSF OR Molgramostin OR Sargramostim | 3 227 |
| **#3** | **#1 AND #2** | 49 |
| **#4** | **#3*** | **48** |

*filters: register of clinical trials

**Supplementary Table 3. EMBASE – last search: 12.02.2026.**

| **Number** | **Keywords** | **Search results** |
| --- | --- | --- |
| **#1** | 'neuroblastoma'/exp OR neuroblastoma OR 'nbl'/exp OR nbl OR neuroblastomas OR 'ganglioneuroblastoma'/exp OR ganglioneuroblastoma OR 'esthesioneuroblastoma'/exp OR esthesioneuroblastoma | 80 319 |
| **#2** | 'granulocyte-macrophage colony-stimulating factor'/exp OR 'granulocyte-macrophage colony-stimulating factor' OR ('granulocyte macrophage' AND 'colony stimulating' AND factor) OR 'granulocyte macrophage colony stimulating factor'/exp OR 'granulocyte macrophage colony stimulating factor' OR (('granulocyte'/exp OR granulocyte) AND ('macrophage'/exp OR macrophage) AND ('colony'/exp OR colony) AND stimulating AND factor) OR 'gm csf'/exp OR 'gm csf' OR (('gm'/exp OR gm) AND ('csf'/exp OR csf)) OR 'csf gm' OR 'histamine-producing cell-stimulating factor' OR ('histamine producing' AND 'cell stimulating' AND factor) OR 'histamine producing cell stimulating factor' OR (('histamine'/exp OR histamine) AND producing AND ('cell'/exp OR cell) AND stimulating AND factor) OR 'gm colony-stimulating factor' OR (('gm'/exp OR gm) AND 'colony stimulating' AND factor) OR 'csf 2' OR 'tc gm csf' OR molgramostin OR 'sargramostim'/exp OR sargramostim | 76 647 |
| **#3** | **#1 AND #2** | 710 |
| **#4** | **#3*** | 208 |

* HUMANS AND [embase]/lim NOT [medline]/lim

**Supplementary Table 4. Other sources –last search: 12.02.2026.**

| **Website** | **Keywords** | **Search results** |
| --- | --- | --- |
| **American Society of Clinical Oncology (ASCO) https://www.asco.org/** | Neuroblastoma | 611 |
| **European Society for Medical Oncology (ESMO) https://www.esmo.org/** | Neuroblastoma | 7 |
| **International Society of Paediatric Oncology (SIOP)**  **https://siop-online.org/** | Neuroblastoma | 47 |

**Supplementary Table 6. PRISMA checklist.**

| **Section and topic** | **Item #** | **Checklist item** | **Location where item is reported** |
| --- | --- | --- | --- |
| **TITLE** | | | |
| Title | **1** | Identify the report as a systematic review. | Title page. |
| **ABSTRACT** | | | |
| Abstract | **2** | See the PRISMA 2020 for Abstracts checklist. | Abstract. |
| **INTRODUCTION** | | | |
| Rationale | **3** | Describe the rationale for the review in the context of existing knowledge. | Introduction, paragraphs 1-8. |
| Objectives | **4** | Provide an explicit statement of the objective(s) or question(s) the review addresses. | Introduction, final paragraph. |
| **METHODS** | | | |
| Eligibility criteria | **5** | Specify the inclusion and exclusion criteria for the review and how studies were grouped for the syntheses. | Materials and methods, eligibility-criteria paragraph; Table 1. |
| Information sources | **6** | Specify all databases, registers, websites, organisations, reference lists and other sources searched or consulted to identify studies. Specify the date when each source was last searched or consulted. | Materials and methods, first paragraph; Supplementary Tables 1-4. |
| Search strategy | **7** | Present the full search strategies for all databases, registers and websites, including any filters and limits used. | Supplementary Tables 1-4. |
| Selection process | **8** | Specify the methods used to decide whether a study met the inclusion criteria of the review, including how many reviewers screened each record and each report retrieved, whether they worked independently, and if applicable, details of automation tools used in the process. | Materials and methods, study-selection paragraph; Figure 1. |
| Data collection process | **9** | Specify the methods used to collect data from reports, including how many reviewers collected data from each report, whether they worked independently, any processes for obtaining or confirming data from study investigators, and if applicable, details of automation tools used in the process. | Materials and methods, data-extraction paragraph. |
| Data items | **10a** | List and define all outcomes for which data were sought. Specify whether all results that were compatible with each outcome domain in each study were sought (e.g. for all measures, time points, analyses), and if not, the methods used to decide which results to collect. | Materials and methods, data-extraction paragraph; Table 1 (Outcomes). |
| Data items | **10b** | List and define all other variables for which data were sought (e.g. participant and intervention characteristics, funding sources). Describe any assumptions made about any missing or unclear information. | Materials and methods, data-extraction paragraph; Supplementary Tables 8-9. |
| Study risk of bias assessment | **11** | Specify the methods used to assess risk of bias in the included studies, including details of the tool(s) used, how many reviewers assessed each study and whether they worked independently, and if applicable, details of automation tools used in the process. | Materials and methods, risk-of-bias paragraph; Supplementary Table 5. |
| Effect measures | **12** | Specify for each outcome the effect measure(s) (e.g. risk ratio, mean difference) used in the synthesis or presentation of results. | Not applicable to quantitative synthesis. No valid component-specific effect estimate could be calculated because the eligible studies did not compare otherwise equivalent anti-GD2 regimens with and without GM-CSF. Arm-level outcome proportions and survival estimates were therefore presented descriptively. |
| Synthesis methods | **13a** | Describe the processes used to decide which studies were eligible for each synthesis (e.g. tabulating the study intervention characteristics and comparing against the planned groups for each synthesis (item #5)). | Materials and methods, eligibility-criteria and synthesis paragraphs; Table 1; Results, study-selection subsection. |
| Synthesis methods | **13b** | Describe any methods required to prepare the data for presentation or synthesis, such as handling of missing summary statistics, or data conversions. | Materials and methods, final paragraph; Results; Supplementary Table 10, footnote d. |
| Synthesis methods | **13c** | Describe any methods used to tabulate or visually display results of individual studies and syntheses. | Materials and methods, final paragraph; Results; Supplementary Tables 8-12; Figures 1 and 2. |
| Synthesis methods | **13d** | Describe any methods used to synthesize results and provide a rationale for the choice(s). If meta-analysis was performed, describe the model(s), method(s) to identify the presence and extent of statistical heterogeneity, and software package(s) used. | Materials and methods, final paragraph. Results were synthesized descriptively because a valid component-specific quantitative synthesis was not feasible. |
| Synthesis methods | **13e** | Describe any methods used to explore possible causes of heterogeneity among study results (e.g. subgroup analysis, meta-regression). | Not applicable. No formal subgroup analysis or meta-regression was conducted because only two clinically and methodologically non-equivalent studies were included and no quantitative synthesis was possible. Sources of heterogeneity were described qualitatively. |
| Synthesis methods | **13f** | Describe any sensitivity analyses conducted to assess robustness of the synthesized results. | Not applicable. No sensitivity analysis was conducted because no quantitative synthesis or component-specific effect estimate was possible. |
| Reporting bias assessment | **14** | Describe any methods used to assess risk of bias due to missing results in a synthesis (arising from reporting biases). | No separate synthesis-level assessment of reporting bias was undertaken because only two heterogeneous studies were included and no quantitative synthesis was performed. Within-study selective reporting was evaluated in ROBINS-I domain 7. |
| Certainty assessment | **15** | Describe any methods used to assess certainty (or confidence) in the body of evidence for an outcome. | No formal certainty-of-evidence assessment was undertaken. Outcome-specific risk of bias was assessed using ROBINS-I, but this was not treated as a certainty-of-evidence assessment. |
| **RESULTS** | | | |
| Study selection | **16a** | Describe the results of the search and selection process, from the number of records identified in the search to the number of studies included in the review, ideally using a flow diagram. | Results, 'Search results and general information about included studies'; Figure 1. |
| Study selection | **16b** | Cite studies that might appear to meet the inclusion criteria, but which were excluded, and explain why they were excluded. | Results, 'Search results and general information about included studies'; Supplementary Table 7. |
| Study characteristics | **17** | Cite each included study and present its characteristics. | Results, 'Search results and general information about included studies'; Supplementary Tables 8-9. |
| Risk of bias in studies | **18** | Present assessments of risk of bias for each included study. | Results, 'Search results and general information about included studies'; Supplementary Table 5. |
| Results of individual studies | **19** | For all outcomes, present, for each study: (a) summary statistics for each group (where appropriate) and (b) an effect estimate and its precision (e.g. confidence/credible interval), ideally using structured tables or plots. | Results, 'Efficacy analysis from included studies' and 'Safety analysis from included studies'; Supplementary Tables 10-12. |
| Results of syntheses | **20a** | For each synthesis, briefly summarise the characteristics and risk of bias among contributing studies. | Results, 'Search results and general information about included studies'; Supplementary Tables 5 and 8-12. |
| Results of syntheses | **20b** | Present results of all statistical syntheses conducted. If meta-analysis was done, present for each the summary estimate and its precision (e.g. confidence/credible interval) and measures of statistical heterogeneity. If comparing groups, describe the direction of the effect. | No statistical synthesis was conducted because the two included studies were clinically and methodologically heterogeneous and did not provide valid component-specific effect estimates. Results were presented descriptively. |
| Results of syntheses | **20c** | Present results of all investigations of possible causes of heterogeneity among study results. | Not applicable. No formal investigation of heterogeneity, such as subgroup analysis or meta-regression, was conducted. Sources of clinical and methodological heterogeneity are described in the Results and Discussion. |
| Results of syntheses | **20d** | Present results of all sensitivity analyses conducted to assess the robustness of the synthesized results. | Not applicable. No sensitivity analysis was conducted because no quantitative synthesis was performed. |
| Reporting biases | **21** | Present assessments of risk of bias due to missing results (arising from reporting biases) for each synthesis assessed. | No separate synthesis-level assessment of reporting bias was undertaken because only two heterogeneous studies were included and no quantitative synthesis was performed. Within-study selective reporting was evaluated in ROBINS-I domain 7; see Supplementary Table 5. |
| Certainty of evidence | **22** | Present assessments of certainty (or confidence) in the body of evidence for each outcome assessed. | No formal certainty-of-evidence assessment was undertaken. The outcome-specific ROBINS-I assessments reported in Supplementary Table 5 concern risk of bias in the included studies and should not be interpreted as a formal certainty-of-evidence assessment. |
| **DISCUSSION** | | | |
| Discussion | **23a** | Provide a general interpretation of the results in the context of other evidence. | Discussion, opening and interpretive paragraphs. |
| Discussion | **23b** | Discuss any limitations of the evidence included in the review. | Discussion, limitations paragraph. |
| Discussion | **23c** | Discuss any limitations of the review processes used. | Materials and methods, eligibility-criteria paragraph and Table 1 footnote; Discussion, limitations paragraph. |
| Discussion | **23d** | Discuss implications of the results for practice, policy, and future research. | Discussion, 'Clinical implications'; Conclusions. |
| **OTHER INFORMATION** | | | |
| Registration and protocol | **24a** | Provide registration information for the review, including register name and registration number, or state that the review was not registered. | Materials and methods, first paragraph (PROSPERO registration CRD420251116911). |
| Registration and protocol | **24b** | Indicate where the review protocol can be accessed, or state that a protocol was not prepared. | No separate protocol document was prepared. The review methods were prospectively registered in PROSPERO (CRD420251116911). |
| Registration and protocol | **24c** | Describe and explain any amendments to information provided at registration or in the protocol. | One post hoc amendment to the prespecified eligibility criteria was made. Frost et al. included 31 patients with neuroblastoma and 2 patients with osteogenic sarcoma. Because the non-neuroblastoma cases could not be fully separated from all pooled outcomes, the study was retained only as indirect regimen-level evidence. This amendment is reported in the Materials and methods, Table 1 footnote, and Discussion. |
| Support | **25** | Describe sources of financial or non-financial support for the review, and the role of the funders or sponsors in the review. | Funding. |
| Competing interests | **26** | Declare any competing interests of review authors. | Conflicts of Interest. |
| Availability of data, code and other materials | **27** | Report which of the following are publicly available and where they can be found: template data collection forms; data extracted from included studies; data used for all analyses; analytic code; any other materials used in the review. | Availability of data and material. All extracted and analyzed data are reported in the article and Supplementary Information; references to the included studies are provided in the bibliography. Additional review materials, including the data-extraction form, are available from the corresponding author on reasonable request. No analytic code was generated because no statistical synthesis was performed. |

*Checklist wording follows PRISMA 2020 (Page MJ et al. BMJ. 2021;372:n71).*

**Supplementary Table 7. Selected recent non-comparative studies evaluating GM-CSF-containing anti-GD2 regimens in neuroblastoma**

| **Study** | **Population** | **Regimen** | **Main results** | **Reason for exclusion** |
| --- | --- | --- | --- | --- |
| Mora 2025 [18] | R/R HR-NBL | Naxitamab + GM-CSF | Reported durable responses and long-term survival in selected patients | No comparator arm without GM-CSF |
| Yu 2025 [39] | Newly diagnosed HR-NBL (n=51) | Dinutuximab beta + GM-CSF + isotretinoin ± VIT chemotherapy | 2-year EFS, 80.1%; 2-year OS, 97.6%; ORR, 60.6% among 33 patients with evidence of disease | No comparator arm without GM-CSF |
| Gong 2026 [40] | R/R HR-NBL | DB + chemotherapy + GM-CSF + isotretinoin | Reported outcomes after more than five cycles of dinutuximab beta-based chemo-immunotherapy | No comparator arm without GM-CSF |
| Kushner 2025 [41] | Primary refractory HR-NBL | Naxitamab + GM-CSF | Reported encouraging survival outcomes in a primary refractory population | No comparator arm without GM-CSF |
| Kushner 2026 [42] | CR2+ patients | Naxitamab + GM-CSF | Reported favorable outcomes in patients in second or later complete remission | No comparator arm without GM-CSF |
| Zhao 2026 [48] | HR-NBL maintenance | DB + GM-CSF + isotretinoin | Reported favorable outcomes with dinutuximab beta combined with GM-CSF and isotretinoin ± chemotherapy as first-line maintenance therapy | No comparator arm without GM-CSF |

DB – dinutuximab beta; R/R – relapsed/refractory; HR-NBL – high-risk neuroblastoma; CR2+ – second or subsequent complete remission; VIT – vincristine, irinotecan, and temozolomide; EFS – event-free survival; OS – overall survival; ORR – objective response rate.
