# Supplementary material for: Comparative evidence on the independent contribution of granulocyte-macrophage colony-stimulating factor to anti-GD2 immunotherapy in neuroblastoma: a systematic review and gap analysis
Source: Front Oncol. 2026 Sep 4;16:1932343. doi: 10.3389/fonc.2026.1932343 (PMC13585510; doi:10.3389/fonc.2026.1932343)
Supplement: Supplementary file 2 [file Table2.docx]

# Supplementary Table 5. ROBINS-I assessment of included non-randomized studies

**Assessment framework:** original ROBINS-I (2016). Target effect: assignment to GM-CSF in addition to an otherwise equivalent anti-GD2 regimen versus the same regimen without GM-CSF. Follow-up began at initiation of anti-GD2 immunotherapy. Judgments were made separately for each outcome.

**Important interpretation.** “Critical” refers to the validity of a causal estimate of the independent GM-CSF effect. It does not imply that the publications lack descriptive value; their results may be reported as indirect regimen-level evidence but should not be synthesized as estimates of a GM-CSF-specific treatment effect.

## A. Summary of domain-level judgments

| **Study** | **Outcome** | **D1** | **D2** | **D3** | **D4** | **D5** | **D6** | **D7** | **Overall** |
| --- | --- | --- | --- | --- | --- | --- | --- | --- | --- |
| **Cheung et al. (2012)** | Progression-free survival | **Critical** | Low | Low | Moderate | Low | Moderate | Moderate | **Critical** |
| **Cheung et al. (2012)** | Overall survival | **Critical** | Low | Low | Moderate | Low | Low | Moderate | **Critical** |
| **Cheung et al. (2012)** | Reported grade 3–4 toxicity frequencies | **Critical** | **Serious** | Low | Moderate | Low | **Critical** | Moderate | **Critical** |
| **Frost et al. (1997)** | Tumor response / antitumor activity | **Critical** | Moderate | Low | **Serious** | **Serious** | Moderate | **Serious** | **Critical** |
| **Frost et al. (1997)** | Toxicity / tolerability | **Critical** | Moderate | Low | **Serious** | Low | Moderate | Moderate | **Critical** |

D1, bias due to confounding; D2, bias in selection of participants into the study; D3, bias in classification of interventions; D4, bias due to deviations from intended interventions; D5, bias due to missing data; D6, bias in measurement of outcomes; D7, bias in selection of the reported result. Categories: Low, Moderate, Serious, Critical, or No information.

## B. Supporting rationale for each judgment

## Cheung et al. (2012) — progression-free survival

**Predicted direction of bias:** *Unpredictable. Later-era care may favor the GM-CSF-containing regimens, whereas the ultra-high-risk subgroup in Regimen C may bias in the opposite direction.*

| **Domain** | **ROBINS-I domain** | **Judgment** | **Supporting rationale** |
| --- | --- | --- | --- |
| **D1** | Bias due to confounding | **Critical** | GM-CSF exposure was inseparable from calendar era and from major baseline treatment and prognostic differences. GM-CSF was introduced with 13-cis-retinoic acid (CRA); induction or salvage therapy, stem-cell transplantation (SCT), baseline risk, 3F8 delivery, supportive care, and post-relapse treatment also differed. Adjustment for selected covariates could not identify an independent GM-CSF effect. |
| **D2** | Bias in selection of participants into the study | **Low** | Patients entered consecutive protocols after achieving complete response or very good partial response and before the assessed anti-GD2 regimen began; survival follow-up started at anti-GD2 initiation. Conditioning on remission limits applicability but does not itself create selection bias for the effect within the enrolled population. |
| **D3** | Bias in classification of interventions | **Low** | Protocol-defined regimens and GM-CSF presence, route, and dose were recorded at treatment initiation; material exposure misclassification is unlikely. |
| **D4** | Bias due to deviations from intended interventions | **Moderate** | Baseline regimen differences are addressed in D1 and were not counted again here. After treatment initiation, early HAMA could delay 3F8 administration, and regimen-specific information on delays, dose completion, and adherence was incomplete. These documented or insufficiently characterized post-assignment changes create some concern, but no major unplanned co-intervention was demonstrated. |
| **D5** | Bias due to missing data | **Low** | Progression-free survival was reported for the full analyzed cohort, with no evidence of material differential loss to follow-up or outcome exclusion. |
| **D6** | Bias in measurement of outcomes | **Moderate** | Disease assessments were scheduled at least every 3 months and used clinical and imaging criteria, but assessment was unblinded and spanned two decades. Progression-free survival depends partly on assessment timing and interpretation, allowing some differential measurement bias. |
| **D7** | Bias in selection of the reported result | **Moderate** | The retrospective cross-protocol analysis had no accessible prespecified analysis plan and allowed multiple model, subgroup, and outcome choices. There was no direct evidence of selective suppression sufficient for a Serious judgment. |

**Overall risk of bias: Critical** — driven by the Critical judgment in D1.

## Cheung et al. (2012) — overall survival

**Predicted direction of bias:** *Unpredictable. Important biases may operate in opposing directions.*

| **Domain** | **ROBINS-I domain** | **Judgment** | **Supporting rationale** |
| --- | --- | --- | --- |
| **D1** | Bias due to confounding | **Critical** | GM-CSF exposure was inseparable from calendar era and from major baseline treatment and prognostic differences. GM-CSF was introduced with CRA; induction or salvage therapy, SCT, baseline risk, 3F8 delivery, supportive care, and post-relapse treatment also differed. Adjustment for selected covariates could not identify an independent GM-CSF effect. |
| **D2** | Bias in selection of participants into the study | **Low** | Patients entered consecutive protocols after achieving complete response or very good partial response and before the assessed anti-GD2 regimen began; survival follow-up started at anti-GD2 initiation. Conditioning on remission limits applicability but does not itself create selection bias for the effect within the enrolled population. |
| **D3** | Bias in classification of interventions | **Low** | Protocol-defined regimens and GM-CSF presence, route, and dose were recorded at treatment initiation; material exposure misclassification is unlikely. |
| **D4** | Bias due to deviations from intended interventions | **Moderate** | Baseline regimen differences are addressed in D1 and were not counted again here. After treatment initiation, early HAMA could delay 3F8 administration, and regimen-specific information on delays, dose completion, and adherence was incomplete. These documented or insufficiently characterized post-assignment changes create some concern, but no major unplanned co-intervention was demonstrated. |
| **D5** | Bias due to missing data | **Low** | Overall survival was reported for the full analyzed cohort, and no material differential loss to follow-up was evident. |
| **D6** | Bias in measurement of outcomes | **Low** | Death is an objective outcome and is unlikely to have been differentially misclassified by knowledge of regimen. |
| **D7** | Bias in selection of the reported result | **Moderate** | The retrospective cross-protocol analysis had no accessible prespecified analysis plan and allowed multiple model, subgroup, and outcome choices. There was no direct evidence of selective suppression sufficient for a Serious judgment. |

**Overall risk of bias: Critical** — driven by the Critical judgment in D1.

## Cheung et al. (2012) — reported grade 3–4 toxicity frequencies

**Predicted direction of bias:** *Unpredictable.*

| **Domain** | **ROBINS-I domain** | **Judgment** | **Supporting rationale** |
| --- | --- | --- | --- |
| **D1** | Bias due to confounding | **Critical** | Treatment era, CRA, SCT exposure, 3F8 delivery, supportive care, and patient risk differed across regimens. These differences were inseparable from GM-CSF exposure and could materially influence reported toxicity. |
| **D2** | Bias in selection of participants into the study | **Serious** | The comparable safety analysis included only 24 of 43 Regimen A patients (the N7 subgroup), because only this subgroup had monitoring considered equivalent to that used for Regimens B and C. Entry into the analyzed safety contrast was therefore protocol- and era-dependent and differed by intervention group. |
| **D3** | Bias in classification of interventions | **Low** | Receipt and route of GM-CSF and the other protocol-defined treatments were clearly recorded. |
| **D4** | Bias due to deviations from intended interventions | **Moderate** | Baseline differences in CRA, SCT, and 3F8 delivery are addressed in D1, not D4. Early HAMA could delay antibody treatment, while regimen-specific adherence, delay, and dose-completion data were incomplete; however, no major unplanned post-assignment co-intervention was documented. |
| **D5** | Bias due to missing data | **Low** | For the safety comparison as explicitly defined by the authors—N7 Regimen A (n = 24) versus Regimens B and C—there was no evidence of additional missing grade 3–4 outcome data. The other 19 Regimen A patients were excluded because ascertainment was not comparable; that mechanism is addressed in D2 and D6 rather than counted again as missing outcome data. |
| **D6** | Bias in measurement of outcomes | **Critical** | The authors explicitly stated that only the N7 subset of Regimen A had toxicity monitoring equivalent to that used for Regimens B and C. Because ascertainment differed systematically across historical eras, the reported frequencies cannot support a valid estimate of an independent GM-CSF safety effect. |
| **D7** | Bias in selection of the reported result | **Moderate** | A broad grade 3–4 toxicity table was reported, but no accessible prespecified analysis plan confirmed all intended safety outcomes and time windows. There was no direct evidence of selective suppression. |

**Overall risk of bias: Critical** — driven by the Critical judgments in D1 and D6.

## Frost et al. (1997) — tumor response / antitumor activity

**Predicted direction of bias:** *Unpredictable.*

| **Domain** | **ROBINS-I domain** | **Judgment** | **Supporting rationale** |
| --- | --- | --- | --- |
| **D1** | Bias due to confounding | **Critical** | GM-CSF was used only in a distinct sequential regimen that also differed in 14.G2a and IL-2 exposure. Regimen-specific baseline characteristics were not reported; diagnosis, disease status, and prior therapy were heterogeneous. With no otherwise equivalent non-GM-CSF group and no adjustment, an independent GM-CSF effect was not identifiable. |
| **D2** | Bias in selection of participants into the study | **Moderate** | Participants entered prospectively before treatment, but allocation across sequential regimens and dose levels was incompletely described. Post-treatment exclusions from the response analysis are addressed in D5. |
| **D3** | Bias in classification of interventions | **Low** | Regimens and the doses and schedules of 14.G2a, IL-2, and GM-CSF were prospectively specified and clearly documented. |
| **D4** | Bias due to deviations from intended interventions | **Serious** | Planned GM-CSF escalation to 125 and 250 µg/m²/day and further accrual to Regimen C were halted after an external neurologic safety signal, despite no within-study GM-CSF dose-limiting toxicity having been reached. The intended escalation strategy was therefore not delivered. Baseline antibody and IL-2 differences are addressed in D1 and are not counted again here. |
| **D5** | Bias due to missing data | **Serious** | Only 26 of 33 treated patients completed one full course and were evaluated for clinical response. Non-evaluation was plausibly related to toxicity, progression, or treatment discontinuation, and no analysis addressed the resulting missing response data. |
| **D6** | Bias in measurement of outcomes | **Moderate** | Standard response criteria were stated, but assessments were unblinded in a small, heterogeneous early-phase population. The measurements are usable descriptively but not robust for a between-regimen causal contrast. |
| **D7** | Bias in selection of the reported result | **Serious** | The trial was designed for dose finding and toxicity, not a GM-CSF efficacy comparison. Responses were largely pooled across regimens, selected individual responders were described, and no prespecified comparative analysis estimated a GM-CSF-specific effect. |

**Overall risk of bias: Critical** — driven by the Critical judgment in D1.

## Frost et al. (1997) — toxicity / tolerability

**Predicted direction of bias:** *Unpredictable.*

| **Domain** | **ROBINS-I domain** | **Judgment** | **Supporting rationale** |
| --- | --- | --- | --- |
| **D1** | Bias due to confounding | **Critical** | GM-CSF was present only in a distinct sequential regimen that also differed in antibody and IL-2 exposure. The small groups differed in disease and treatment history, regimen-specific baseline characteristics were not reported, and the comparison was unadjusted. An independent GM-CSF safety effect was not identifiable. |
| **D2** | Bias in selection of participants into the study | **Moderate** | Patients were enrolled prospectively before treatment, but the process assigning eligible patients to sequential regimens and dose levels was incompletely described. |
| **D3** | Bias in classification of interventions | **Low** | Treatment components, doses, and schedules were prospectively defined and recorded, making intervention misclassification unlikely. |
| **D4** | Bias due to deviations from intended interventions | **Serious** | Planned GM-CSF dose escalation and further accrual to Regimen C were administratively halted at the second level after an external neurologic safety signal, despite no within-study GM-CSF dose-limiting toxicity. Consequently, the intended GM-CSF exposure range was not delivered. Baseline antibody and IL-2 differences are addressed in D1. |
| **D5** | Bias due to missing data | **Low** | All 33 treated patients were monitored for toxicity. Patients who discontinued during a course were retained in the course-based toxicity summaries, and withdrawals and their reasons were reported. There was no evidence of material missing data for the core toxicity outcome; HAMA was an immunogenicity outcome and was not used to judge this domain. |
| **D6** | Bias in measurement of outcomes | **Moderate** | Toxicities were prospectively collected with specified biologic-therapy grading scales and reported to the CCG and NCI, but the study was open label, causal attribution was clinician judged, and exposure and course counts differed across regimens. |
| **D7** | Bias in selection of the reported result | **Moderate** | Dose-limiting and grade 2–4 toxicities were central outcomes and were reported, but no accessible analysis plan excluded selective choice among dose-level, regimen-level, and per-course summaries. |

**Overall risk of bias: Critical** — driven by the Critical judgment in D1.

## C. Methodological notes

- The original seven-domain ROBINS-I tool (2016) was used. ROBINS-I V2 remained a draft at the time this table was prepared.
- The target effect was the effect of assignment to GM-CSF in addition to an otherwise equivalent anti-GD2 regimen. Planned baseline differences between historical regimen bundles were assessed in D1. D4 was reserved for post-assignment deviations or failures to deliver the intended intervention strategy.
- Progression-free survival and overall survival were assessed separately because death is objective, whereas progression may be affected by assessment timing and lack of blinding.
- The Cheung safety result was defined as the reported comparison of the N7 subset of Regimen A (n = 24) with Regimens B and C. The exclusion of the other 19 Regimen A patients because of non-comparable monitoring was evaluated in D2 and D6, not counted again in D5.
- For Frost toxicity, HAMA was treated as an immunogenicity outcome, not as toxicity. All 33 treated participants contributed to toxicity monitoring; treatment withdrawals were retained in the course-based toxicity summaries.
- A Critical judgment in any domain yields a Critical overall judgment for the assessed result. All five overall judgments remain Critical because the independent GM-CSF effect was not identifiable from the non-equivalent regimens; the Cheung toxicity result also had Critical risk in D6.

### Abbreviations

3F8, murine anti-GD2 monoclonal antibody 3F8; 14.G2a, murine anti-GD2 monoclonal antibody 14.G2a; CCG, Children’s Cancer Group; CRA, 13-cis-retinoic acid; CR, complete response; DLT, dose-limiting toxicity; GM-CSF, granulocyte-macrophage colony-stimulating factor; HAMA, human anti-mouse antibody; HR, high risk; IL-2, interleukin-2; N7, Memorial Sloan Kettering treatment protocol N7; NCI, National Cancer Institute; OS, overall survival; PFS, progression-free survival; SC, subcutaneous; SCT, stem-cell transplantation; UHR, ultra-high risk; VGPR, very good partial response.

### References

1. Sterne JAC, Hernán MA, Reeves BC, Savović J, Berkman ND, Viswanathan M, Henry D, Altman DG, Ansari MT, Boutron I, Carpenter JR, Chan AW, Churchill R, Deeks JJ, Hróbjartsson A, Kirkham J, Jüni P, Loke YK, Pigott TD, Ramsay CR, Regidor D, Rothstein HR, Sandhu L, Santaguida PL, Schünemann HJ, Shea B, Shrier I, Tugwell P, Turner L, Valentine JC, Waddington H, Waters E, Wells GA, Whiting PF, Higgins JPT. ROBINS-I: a tool for assessing risk of bias in non-randomised studies of interventions. BMJ. 2016;355:i4919. doi:10.1136/bmj.i4919.

2. Cheung NKV, Cheung IY, Kushner BH, Ostrovnaya I, Chamberlain E, Kramer K, Modak S. Murine anti-GD2 monoclonal antibody 3F8 combined with granulocyte-macrophage colony-stimulating factor and 13-cis-retinoic acid in high-risk patients with stage 4 neuroblastoma in first remission. J Clin Oncol. 2012;30(26):3264–3270. doi:10.1200/JCO.2011.41.3807.

3. Frost JD, Hank JA, Reaman GH, Frierdich S, Seeger RC, Gan J, Anderson PM, Ettinger LJ, Cairo MS, Blazar BR, Krailo MD, Matthay KK, Reisfeld RA, Sondel PM. A phase I/IB trial of murine monoclonal anti-GD2 antibody 14.G2a plus interleukin-2 in children with refractory neuroblastoma: a report of the Children’s Cancer Group. Cancer. 1997;80(2):317–333. doi:10.1002/(SICI)1097-0142(19970715)80:2<317::AID-CNCR21>3.0.CO;2-W.
