# Supplementary material for: Comparative evidence on the independent contribution of granulocyte-macrophage colony-stimulating factor to anti-GD2 immunotherapy in neuroblastoma: a systematic review and gap analysis
Source: Front Oncol. 2026 Sep 4;16:1932343. doi: 10.3389/fonc.2026.1932343 (PMC13585510; doi:10.3389/fonc.2026.1932343)
Supplement: Supplementary file 3 [file Table3.docx]

**Supplementary Tables 8–12**

**Supplementary Table 8. Methodological characteristics of studies comparing anti-GD2 therapy with and without GM-CSF in patients with neuroblastoma**

| **Reference** | **Type of trial** | **Population** | **Assessed interventions and number of included patients** | **Follow-up** |
| --- | --- | --- | --- | --- |
| **Cheung et al. 2012 [37]** | Open-label, single-center, comparative study of retrospective data, multi-arm | Children with stage 4 neuroblastoma who underwent chemotherapy based on the Memorial Sloan-Kettering Cancer Center or COG3973 induction, hyperfractionated 21-Gy local radiation, and surgical resection of the primary tumor, with or without second-line therapy to achieve remission, and with or without myeloablative therapy with stem cell rescue. After confirmed CR/VGPR according to the International  Neuroblastoma Response Criteria, institutional review board–approved protocols were applied. Ultra–high-risk patients had refractory disease, requiring additional cyclophosphamide ± topotecan or irinotecan after standard induction therapy to achieve CR/VGPR before enrollment for immunotherapy. | Regimen A: m3F8 [year 1988 to 2000]; high-risk patients (n=43)  Regimen B: m3F8 in combination with IV GM-CSF and oral 13-*cis*-RA [year 1999 to 2003]; high-risk patients (n=41)  Regimen C: m3F8 in combination with SC GM-CSF and oral 13-*cis*-RA [year 2003 to 2008]; n=85 (including 57 high-risk patients and 28 ultra–high-risk patients).  GM-CSF doses were increased from 250 to 500 µg/m^2^ in each patient after the second day of m3F8 administration, provided the absolute neutrophil count was ≤20,000/µL. Treatment cycles were separated by rest periods of 2 to 4 weeks for the first 4 cycles, and of 6 to 8 weeks thereafter, continuing up to 24 months from study entry or until progressive disease, whichever occurred first. For patients with elevated human anti-mouse antibody titer, treatment was deferred until titers became negative. Starting after the second cycle of immunotherapy, oral 13-*cis*-RA was initiated and administered for 6 cycles between courses of m3F8+GM-CSF. | All progression-free patients had a minimum follow-up duration of 2.9 years from the initiation of immunotherapy and of 3.6 years from diagnosis. |
| **Frost et al. 1997 [38]** | Open-label, multicenter (12 institutions), prospective, phase I/Ib, multi-arm trial | Patients under 21 years of age at the time of initial biopsy-proven diagnosis of neuroblastoma or osteogenic sarcoma were eligible for enrollment. Additionally, patients with other GD2-positive tumors at initial diagnosis, such as primitive neuroectodermal tumors and osteogenic sarcoma, were also eligible to participate.  Eligible patients had to meet the following criteria: failure to achieve a CR following aggressive chemotherapy, including autologous BMT; progressive disease after aggressive chemotherapy; or partial response to reinduction therapy with a high likelihood of subsequent tumor progression. All patients were required to have received at least one prior multi-agent chemotherapeutic regimen before study entry. Additional eligibility criteria included completion of all radiotherapy, cytotoxic, or biologic therapies at least 2 weeks prior to enrollment; a life expectancy of at least 3 months; an ECOG performance status of 0-2; and adequate liver, cardiac, renal, hematologic, and central nervous system function. Patients with prior in vivo biologic therapy involving murine-derived antibodies were excluded. | Regimen A: determination of the MTD of 14.G2a when administered with IL-2. Seven groups of patients to receive escalating 14.G2a doses in combination with IL-2 (3 patients in each group): 14.G2a at 2, 10, 15, 20, 30, 40, and 60 mg/m2/day (as a 2-hour intravenous infusion) for 5 consecutive days on days 9-13 of the treatment course. IL-2 at a dose of 3.0 x10^6^ U/m^2^/day IV by continuous infusion for 96 hours on days 1–4, 8–11, and 15–18 of each course.  Total number of patients: 18.  Regimen B: delineation of toxicities specific to 14.G2a without IL-2 at the MTD determined in Regimen A. The MoAb was given on days 1-5 at a dose of 15 mg/m^2^/day without concurrent IL-2. During weeks 5–10, all patients received the 14.G2a with IL-2 as described for Regimen A.  Total number of patients: 8.  Regimen C: evaluation of toxicities caused by escalating GM-CSF doses added to the MoAb and IL-2 dosing schedule. Four dose combinations were scheduled, with 3 patients evaluated in each dose combination. Patients followed the same 14.G2a and IL-2 schedule as for Regimen A, at 15 mg/m^2^/day of 14.G2a, with the addition of SC GM-CSF on days 1–19. In the first group, IL-2 was administered at 1.5 x10^6^ U/m^2^/day and GM-CSF at 62.5 µg/m^2^/ day, one-fourth the standard myelostimulatory dose. In the second group, IL-2 was increased to 3.0x10^6^ U/m^2^/day, while 14.G2a and GM-CSF doses remained unchanged. Dose escalation to 125 and 250 µg/m^2^/day of GM-CSF were planned for the third and fourth groups of patients, with IL-2 and 14.G2a at the same doses.  Total number of patients: 7. | Outcomes assessed during treatment; no reported mean or median post-treatment  follow-up |

BMT – bone marrow transplantation; CR – complete response, GM-CSF – granulocyte-macrophage colony stimulating factor; IL-2 – interleukin 2; IV – intravenous; MoAb – monoclonal antibody; MTD – maximum tolerated dose; SC – subcutaneous; VGPR – very good partial response; 13-*cis*-RA – 13-*cis* retinoic acid

**Supplementary Table 9. Patient and treatment characteristics of the included studies.**

**A. Demographic and disease characteristics**

| **Reference** | **Study group** | **Age** | **Male, n/N (%)** | **MYCN amplified, n/N (%)** | **LDH >1,500 U/mL, n/N (%)** | **Bone involvement, n/N (%)** | **Bone marrow involvement, n/N (%)** |
| --- | --- | --- | --- | --- | --- | --- | --- |
| Cheung et al. 2012 [37] | Regimen A (m3F8), n=43 | ≥18 months at diagnosis: 41/43 (95%) | 25/43 (58%) | 15/43 (35%) | 14/43 (33%) | 32/43 (74%) | 39/43 (91%) |
|  | Regimen B (m3F8 + IV GM-CSF + 13-cis-RA), n=41 | ≥18 months at diagnosis: 38/41 (93%) | 26/41 (63%) | 14/41 (34%) | 15/41 (37%) | 31/41 (76%) | 33/41 (80%) |
|  | Regimen C (m3F8 + SC GM-CSF + 13-cis-RA), HR, n=57 | ≥18 months at diagnosis: 52/57 (91%) | 39/57 (68%) | 29/57 (51%) | 18/57 (32%) | 42/57 (74%) | 50/57 (88%) |
|  | Regimen C (m3F8 + SC GM-CSF + 13-cis-RA), UHR, n=28 | ≥18 months at diagnosis: 27/28 (96%) | 12/28 (43%) | 7/28 (25%) | 4/28 (14%) | 17/28 (61%) | 23/28 (82%) |
| Frost et al. 1997 [38] | All treated patients, n=33 (31 with neuroblastoma; 2 with osteogenic sarcoma)ᵃ | At diagnosis: mean 5.7 years; median 9.2 years; range 1.6–16.7 years At study entry: mean 7.7 years; median 9.9 years; range 2.6–17.2 years | 18/33 (54.5%) | NR | NR | NR | NR |

ᵃ Frost et al. did not report patient characteristics separately by treatment regimen.

GM-CSF – granulocyte-macrophage colony-stimulating factor; HR – high risk; IV – intravenous; LDH – lactate dehydrogenase; m3F8 – murine anti-GD2 monoclonal antibody 3F8; NR – not reported; SC – subcutaneous; UHR – ultra-high risk; 13-cis-RA – 13-cis-retinoic acid.

**B. Prior treatment and treatment-related characteristics**

| **Reference** | **Study group** | **Prior SCT exposure, n/N (%)** | **HAMA response, n/N assessed (%)** | **Induction: MSKCC–COG3973 regimen, n/N (%)** | **Induction: intensive non–MSKCC–COG3973 regimen, n/N (%)** | **Induction: less intensive regimen, n/N (%)** | **13-cis-RA, n/N (%)** |
| --- | --- | --- | --- | --- | --- | --- | --- |
| Cheung et al. 2012 [37] | Regimen A (m3F8), n=43 | 1/43 (2%) | 24/43 (56%) | 43/43 (100%) | 0/43 (0%) | 0/43 (0%) | 6/43 (14%) |
|  | Regimen B (m3F8 + IV GM-CSF + 13-cis-RA), n=41 | 40/41 (98%) | 29/41 (71%) | 28/41 (69%) | 3/41 (7%) | 10/41 (24%) | 37/41 (90%) |
|  | Regimen C (m3F8 + SC GM-CSF + 13-cis-RA), HR, n=57 | 37/57 (65%) | 46/57 (80%) | 50/57 (87%) | 5/57 (9%) | 2/57 (4%) | 53/57 (93%) |
|  | Regimen C (m3F8 + SC GM-CSF + 13-cis-RA), UHR, n=28 | 9/28 (32%) | 21/28 (75%) | 22/28 (78%) | 3/28 (11%) | 3/28 (11%) | 28/28 (100%) |
| Frost et al. 1997 [38] | All treated patients, n=33 (31 with neuroblastoma; 2 with osteogenic sarcoma)ᵃ | TBI/autologous BMT: 10/33 (30.3%) | 9/21 evaluated (42.9%)ᵇ | NR | NR | NR | NR |

ᵃ Frost et al. did not report prior treatment or treatment-related characteristics separately by treatment regimen.

ᵇ Frost et al. reported human anti-mouse antibodies in 9 of 21 evaluated patients; because antibody-response definitions and ascertainment differed from those used by Cheung et al., this value should not be interpreted as directly comparable.

BMT – bone marrow transplantation; COG – Children’s Oncology Group; GM-CSF – granulocyte-macrophage colony-stimulating factor; HAMA – human anti-mouse antibody; HR – high risk; IV – intravenous; MSKCC – Memorial Sloan Kettering Cancer Center; m3F8 – murine anti-GD2 monoclonal antibody 3F8; NR – not reported; SC – subcutaneous; SCT – stem-cell transplantation; TBI – total-body irradiation; UHR – ultra-high risk; 13-cis-RA – 13-cis-retinoic acid.

**Supplementary Table 10. Key efficacy results of the included studies.**

| **Reference** | **Study group** | **5-year PFS, % [95% CI]** | **5-year OS, % [95% CI]** | **Median time to relapse or death from immunotherapy initiation, years** | **Patients without recorded relapse, nᵇ** | **Median follow-up among patients without recorded relapse, yearsᵇ** | **Patients experiencing relapse, %** | **Response findings** |
| --- | --- | --- | --- | --- | --- | --- | --- | --- |
| Cheung et al. 2012 [37]ᵃ | Regimen A (m3F8), HR, n=43 | 44 [32–62] | 49 [36–66] | 2.7 | 20 | 15.5 | 53 | NA: patients entered immunotherapy in CR/VGPR |
|  | Regimen B (m3F8 + IV GM-CSF + 13-cis-RA), HR, n=41 | 56 [43–74] | 61 [48–78] | Not reached | 24 | 10.2 | 41 |  |
|  | Regimen C (m3F8 + SC GM-CSF + 13-cis-RA), HR, n=57 | Overall: 62 [50–76] No-SCT subgroup (n=20): 50 [32–78] | Overall: 81 [70–92] No-SCT subgroup (n=20): 84 [69–100] | Not reached | 36 | 5.9 | 37 |  |
|  | Regimen C (m3F8 + SC GM-CSF + 13-cis-RA), UHR, n=28ᶜ | 36 [22–59] | 75 [60–93] | 1.5 | 10 | 4.4 | 64 |  |
| Frost et al. 1997 [38] | Pooled study-level results across regimens A–C, n=33 (31 with neuroblastoma; 2 with osteogenic sarcoma); 26 evaluable for response | Not reported | Not reported | Not reported | Not reported | Not reported | Not reported | 9/26 evaluable patients (34.6%; 7 SD, 1 PR, and 1 CR) had no tumor progression during the on-study evaluation period. Four patients with neuroblastoma in regimen A had a clinical or bone-marrow response; one patient with osteogenic sarcoma in regimen B achieved CR 2 months after therapy without additional treatment.ᵈ |

ᵃ Across the four Cheung groups, PFS and OS differed in univariate log-rank analyses (P=0.018 and P=0.003, respectively).

ᵇ Cheung et al., Table 2, labeled these patients as ‘PF patients’. However, three patients in regimen A and one in regimen B died without relapse; therefore, the values 20, 24, 36, and 10 are presented here more precisely as the numbers of patients without recorded relapse. Median follow-up refers to the same source-defined subgroup.

ᶜ UHR patients received additional cyclophosphamide ± topotecan or irinotecan for refractory disease after standard induction therapy to achieve CR/VGPR before m3F8 immunotherapy; these salvage therapies were not available until the regimen C era.

ᵈ Frost et al. reported 27%; using the stated denominator of 26 evaluable patients gives 9/26=34.6% (35% after rounding). The published 27% appears to have been calculated using all 33 treated patients (9/33=27.3%).

CI – confidence interval; CR – complete response; GM-CSF – granulocyte-macrophage colony-stimulating factor; HR – high risk; IV – intravenous; m3F8 – murine anti-GD2 monoclonal antibody 3F8; NA – not applicable; OS – overall survival; PFS – progression-free survival; PR – partial response; SC – subcutaneous; SCT – stem-cell transplantation; SD – stable disease; UHR – ultra-high risk; 13-cis-RA – 13-cis-retinoic acid.

**Supplementary Table 11. Patients with grade 3–4 toxicities across treatment regimens in Cheung et al. (2012) [37].**

| **Grade 3–4 toxicity** | **Regimen A: N7 subgroup (m3F8), n=24; patients (%)ᵃ** | **Regimen B (m3F8 + IV GM-CSF), n=41; patients (%)** | **Regimen C (m3F8 + SC GM-CSF), n=85; patients (%)** |
| --- | --- | --- | --- |
| Painᵇ | 0 | 2 | 1 |
| Hypotension | 0 | 2 | 0 |
| Hypoxiaᶜ | 0 | 5 | 3 |
| Fever without neutropenia | 0 | 12 | 5 |
| Acute capillary leak syndrome | 0 | 0 | 0 |
| Hypersensitivity reactionᵈ | 0 | 0 | 4 |
| Urticariaᵈ | 0 | 0 | 0 |
| Infection | 4 | 5 | 14 |
| Catheter-related infection | 0 | 0 | 1 |
| Nausea | 0 | 2 | 0 |
| Vomiting | 0 | 2 | 2 |
| Diarrhea | 0 | 0 | 4 |
| Hyponatremia | 29 | 7 | 0 |
| Hypokalemia | 29 | 12 | 11 |
| Abnormal ALT | 17 | 7 | 8 |
| Abnormal AST | 8 | 0 | 4 |
| Hypercalcemia | 0 | 0 | 0 |
| Serum sicknessᵈ | 0 | 0 | 0 |
| Seizure | 0 | 0 | 0 |
| CNS cortical syndrome | 0 | 0 | 0 |
| Hypertension | 0 | 5 | 2 |

Values are percentages of patients, not numbers of events. Percentages must not be summed across toxicity categories because individual patients could experience more than one toxicity.

ᵃ Only the 24 patients treated on the N7 protocol within regimen A underwent toxicity monitoring comparable with that used for regimens B and C.

ᵇ All patients at MSKCC were treated as outpatients using a previously described pain-management regimen.

ᶜ One patient receiving regimen C developed idiopathic pneumonitis considered secondary to pulmonary toxicity from autologous transplantation, surgery, and intraoperative radiotherapy; the patient recovered completely.

ᵈ Immunotherapy was administered only to HAMA-negative patients, and all patients received antihistamine premedication. Although urticaria was common, it was always grade ≤2.

Safety comparisons should be interpreted cautiously because toxicity ascertainment differed across treatment eras and only the N7 subgroup of regimen A had monitoring comparable with that used for regimens B and C.

ALT – alanine aminotransferase; AST – aspartate aminotransferase; CNS – central nervous system; GM-CSF – granulocyte-macrophage colony-stimulating factor; HAMA – human anti-mouse antibody; IV – intravenous; m3F8 – murine anti-GD2 monoclonal antibody 3F8; MSKCC – Memorial Sloan Kettering Cancer Center; SC – subcutaneous.

Values represent the reported percentages of patients with each grade 3–4 toxicity. For regimen A, comparable toxicity monitoring was available only for the 24 patients treated under the N7 protocol. Because toxicity ascertainment and recording procedures differed across treatment eras, comparisons between regimens are descriptive only and should not be interpreted as estimates of the independent effect of GM-CSF.

**Supplementary Table 12. Key safety results from Frost et al. (1997) [38].**

| **Outcome** | **Regimen A (14.G2a dose escalation + IL-2)** | **Regimen B (14.G2a at 15 mg/m²/day alone on days 1–5, followed by 14.G2a + IL-2 during weeks 5–10)** | **Regimen C (14.G2a 15 mg/m2/day + IL-2 + GM-CSF)** |
| --- | --- | --- | --- |
| **Dose-limiting toxicity** | At the 20 mg/m²/day dose level, eight patients were enrolled and seven received 14.G2a. Dose-limiting toxicities were observed, and one additional patient withdrew before receiving 14.G2a because of IL-2 toxicity. The MTD of 14.G2a in combination with IL-2 was established at 15 mg/m²/day. | - | Dose escalation of IL-2 and GM-CSF in Regimen C was administratively halted at the second dose level, which included GM-CSF at 62.5 µg/m²/day, IL-2 at 3.0 × 10⁶ U/m²/day, and 14.G2a at 15 mg/m²/day. This decision was made despite the fact that dose-limiting toxicity had not yet been reached for the combination of GM-CSF, IL-2, and 14.G2a. The halt in accrual to this treatment arm was based on concerns arising from newly identified neurologic toxicities reported in a separate study, in which GM-CSF was administered alongside IL-2 in adults with metastatic renal cell carcinoma. These neurologic events coincided with hyperleukocytosis, raising safety concerns about the continued dose escalation in Regimen C. |
| **Grade 2 toxicity (rate/course)** | A (2 mg/m^2^/day; n=3): 5 (1.25)  A (10 mg/m^2^/day; n=4): 7 (1.52)  A (15 mg/m^2^/day; n=3): 12 (2.40)  A (20 mg/m^2^/day; n=8): 17 (2.59) | n=8  23 (1.77) | n=7  26 (2.89) |
| **Grade 3 toxicity (rate/course)** | A (2 mg/m^2^/day; n=3): 10 (2.50)  A (10 mg/m^2^/day; n=4): 6 (1.30)  A (15 mg/m^2^/day; n=3): 13 (2.60)  A (20 mg/m^2^/day; n=8): 15 (2.28) | n=8  14 (1.08) | n=7  31 (3.44) |
| **Grade 4 toxicity (rate/course)** | A (2 mg/m^2^/day; n=3): 2 (0.50)  A (10 mg/m^2^/day; n=4): 5 (1.09)  A (15 mg/m^2^/day; n=3): 2 (0.40)  A (20 mg/m^2^/day; n=8): 11 (1.67) | n=8  5 (0.38) | n=7  2 (0.22) |
| **Grade 4 toxicity used to determine the maximum tolerated dose** | A (2 mg/m^2^/day; n=3): 0  A (10 mg/m^2^/day; n=4): 2  A (15 mg/m^2^/day; n=3): 0  A (20 mg/m^2^/day; n=8): 4 | - | - |

GM-CSF – granulocyte-macrophage colony-stimulating factor; IL-2 – interleukin-2; MTD – maximum tolerated dose. Values before parentheses represent the number of toxicities of the specified grade; values in parentheses represent the estimated number of toxicities per patient-course.
